# Silencing ${\text{I}}_{2}^{\text{PP2A}}$ Rescues Tau Pathologies and Memory Deficits through Rescuing PP2A and Inhibiting GSK-3β Signaling in Human Tau Transgenic Mice

**DOI:** 10.3389/fnagi.2014.00123

**Published:** 2014-06-17

**Authors:** Yao Zhang, Rong-Hong Ma, Xia-Chun Li, Jia-Yu Zhang, Hai-Rong Shi, Wei Wei, Dan-Ju Luo, Qun Wang, Jian-Zhi Wang, Gong-Ping Liu

**Affiliations:** ^1^Key Laboratory of Neurological Disease of Chinese Ministry of Education, Huazhong University of Science and Technology, Wuhan, China; ^2^Department of Endocrinology, Liyuan Hospital, Huazhong University of Science and Technology, Wuhan, China; ^3^Department of Laboratory Medicine, Union Hospital, Tongji Medical College, Huazhong University of Science and Technology, Wuhan, China; ^4^Department of Pathophysiology, Tongji Medical College, Huazhong University of Science and Technology, Wuhan, China; ^5^Department of Pathophysiology, Jinan University, Guangzhou, China

**Keywords:** Alzheimer disease, inhibitor-2 of protein phosphatase-2A, glycogen synthase kinase-3β, tau hyperphosphorylation, memory

## Abstract

Increase of inhibitor-2 of protein phosphatase-2A $\left({\text{I}}_{2}^{\text{PP2A}}\right)$ is associated with protein phosphatase-2A (PP2A) inhibition and tau hyperphosphorylation in Alzheimer’s disease (AD). Down-regulating ${\text{I}}_{2}^{\text{PP2A}}$ attenuated amyloidogenesis and improved the cognitive functions in transgenic mice expressing amyloid precursor protein (tg2576). Here, we found that silencing ${\text{I}}_{2}^{\text{PP2A}}$ by hippocampal infusion of ${\text{Lenti - siI}}_{2}^{\text{PP2A}}$ down-regulated ${\text{I}}_{2}^{\text{PP2A}}$ (~45%) with reduction of tau phosphorylation/accumulation, improvement of memory deficits, and dendritic plasticity in 12-month-old human tau transgenic mice. Silencing ${\text{I}}_{2}^{\text{PP2A}}$ not only restored PP2A activity but also inhibited glycogen synthase kinase-3β (GSK-3β) with a significant activation of protein kinase A (PKA) and Akt. In HEK293/tau and N2a/tau cells, silencing ${\text{I}}_{2}^{\text{PP2A}}$ by ${\text{pSUPER - siI}}_{2}^{\text{PP2A}}$ also significantly reduced tau hyperphosphorylation with restoration of PP2A activity and inhibition of GSK-3β, demonstrated by the decreased GSK-3β total protein and mRNA levels, and the increased inhibitory phosphorylation of GSK-3β at serine-9. Furthermore, activation of PKA but not Akt mediated the inhibition of GSK-3β by ${\text{I}}_{2}^{\text{PP2A}}$ silencing. We conclude that targeting ${\text{I}}_{2}^{\text{PP2A}}$ can improve tau pathologies and memory deficits in human tau transgenic mice, and activation of PKA contributes to GSK-3β inhibition induced by silencing ${\text{I}}_{2}^{\text{PP2A}}$ *in vitro*, suggesting that ${\text{I}}_{2}^{\text{PP2A}}$ is a promising multiple target of AD.

## Introduction

Alzheimer’s disease (AD) is the neurodegenerative disorder. Neuropathological studies have demonstrated that formation of neurofibrillary tangles (NFTs) is one of the most prominent pathologic characteristics in the brain of AD patients, and the abnormally hyperphosphorylated tau is the major protein subunit of the tangles (Grundke-Iqbal et al., 1986; Lee et al., 1991). Though the mechanism leading to the formation of NFTs is still elusive, it has been well recognized that an imbalanced regulation in protein kinases and protein phosphatases is the direct cause for the AD-like tau hyperphosphorylation (Gong et al., 2000; Planel et al., 2000; Liu et al., 2005; Qian et al., 2010). Among various kinases and phosphatases, glycogen synthase kinase-3β (GSK-3β; Avila and Díıaz-Nido, 2004; Takashima, 2006; Hernandez et al., 2013) and protein phosphatase (PP)-2A (Wang et al., 1995; Gong et al., 2000; Liu et al., 2005; Rudrabhatla and Pant, 2011) are the most implicated.

Of the two isoforms of GSK-3, GSK-3α and GSK-3β, GSK-3β is the major tau kinase (Ishiquro et al., 1993; Takashima et al., 1996) which can phosphorylate tau at multiple AD-related sites, including Ser-46, Thr-50, Thr-175, Thr-181, Ser-199, Ser-202, Thr-205, Thr-212, Thr-217, Thr-231, Ser-235, Ser-396, Ser-400, Ser-404, and Ser-413 (Wang and Liu, 2008; Hanger et al., 2009). GSK-3β has also been directly linked to several key pathological mechanisms of AD (Lovestone et al., 1994; Hong et al., 1997; Munoz-Montano et al., 1997; Liu et al., 2003; Cai et al., 2012). In the AD brains, activated GSK-3β is accumulated not only in a subpopulation of neurons with NFTs but also in dystrophic neurites of senile plaques, neuropil threads, Pick bodies, tau-containing astrocytes, and coiled bodies (Pei et al., 1999; Ferrer et al., 2002). It was also observed in transfected cells and rat brains that activation of GSK-3β could efficiently induce tau hyperphosphorylation at most of the hyperphosphorylated sites seen in the paired helical filaments (PHFs) isolated from AD brains (Lovestone et al., 1994; Hong et al., 1997; Liu et al., 2005; Cavallini et al., 2013). Transgenic mice overexpressing GSK-3β display tau hyperphosphorylation, disrupted microtubules, and apoptotic neurons (Lucas et al., 2001). Therefore, downregulation of GSK-3β could be promising in arresting AD pathologies.

Protein phosphatase-2A (PP2A) accounts for ~70% of the total tau phosphatase activity in human brain and the activity of PP2A is significantly inhibited in the AD brains with concurrent hyperphosphorylation of tau (Gong et al., 1993, 1995; Liu et al., 2005). *In vitro* and animal studies have demonstrated that inhibition of PP2A causes tau hyperphosphorylation and the related pathological alterations, while simultaneous upregulation of PP2A rescues the pathologies (Wang et al., 1995, 1998, 2007; Gong et al., 2000; Sun et al., 2003; Tian et al., 2004; Arif et al., 2014). Negative correlation between PP2A activity and the level of tau phosphorylation at most of the phosphorylation sites in human brains further supports the dominant role of PP2A in regulation of tau phosphorylation as compared with other protein phosphatases (Liu et al., 2005). Recent studies demonstrate that PP2A is inhibited by intracellular heat-stable factors namely PP2A inhibitor-2 $\left({\text{I}}_{2}^{\text{PP2A}}\right)$ and inhibitor 1 (Li et al., 1995; Tsujio et al., 2005) and activated by phospho-tyrosyl phosphatase activator (PTPA) (Luo et al., 2013).

In the AD brain, both the transcript and the protein levels of ${\text{I}}_{2}^{\text{PP2A}}$ are increased (Tanimukai et al., 2005), and the ${\text{I}}_{2}^{\text{PP2A}}$ protein is co-localized with PP2A and the abnormally hyperphosphorylated tau in the neuronal cytoplasm (Tanimukai et al., 2005). Silencing ${\text{I}}_{2}^{\text{PP2A}}$ can restore PP2A activity and ameliorate amyloidogenesis in tg2576 mice (Liu et al., 2013). The human tau transgenic mouse (htau) is the ideal model for searching the target to inhibit tau pathologies, however, the effects of ${\text{I}}_{2}^{\text{PP2A}}$ knockdown in this mouse model has not been studied.

In the present study, we found that silencing ${\text{I}}_{2}^{\text{PP2A}}$ could improve tau pathologies with improvement of memory deficits through activation of PP2A and inhibition of GSK-3β in htau mice, further studies in HEK293/tau and N2a/tau cells demonstrate that activation of protein kinase A (PKA) but not Akt mediates the GSK-3β inhibition induced by silencing ${\text{I}}_{2}^{\text{PP2A}}$.

## Materials and Methods

### Antibodies and construction of plasmids

The detailed information for the antibodies used in this work is listed in Table S1 in Supplementary Material. *R*_p_-adenosine 3′, 5′-cyclic monophosphorothioate triethyl ammonium salt (*R*_p_-cAMPS, a specific inhibitor of PKA) and okadaic acid (OA) were purchased from Sigma. To knockdown ${\text{I}}_{2}^{\text{PP2A}}$ in cells, shRNA oligo sequences were synthesized as follow: 5′-AGCTTGGATGAAGGTGAAGAAGATTTCAAGAGAATCTTCTTCACCTTCATCCTTTTTC-3′, 5′-TCGAGAAAAAGGATGAAGGTGAAGAAGATTCTCTTGAAATCTTCTTCACCTTCATCCA-3′. As control, we used non-functional ${\text{I}}_{2}^{\text{PP2A}}$-derived sequences: 5′-AGCTTTGAGAGTGGTGATCCATCTTTCAAGAGAAGATGGATCACCACTCTCATTTTTC-3′, 5′-TCGAGAAAAATGAGAGTGGTGATCCATCTTCTCTTGAAAGATGGATCACCACTCTCAA-3′ (ten Klooster et al., 2007; Liu et al., 2008). All were purchased as 63-nt ssDNA oligomers composed of both forward and reverse sequences with 9-bp loop structures (Brummelkamp et al., 2002) and 3′ *Xho*1 I and 5′ *Hin*dIII self-inactivating overhangs. Sense and antisense oligomers (both at 20 μM) were incubated in annealing buffer for 3 min at 90°C as described (Elbashir et al., 2001), then the temperature was lowered in 2°C/min increments until 5°C above their respective *T*_m_ and then dropped to 4°C at maximum ramp rates. pSUPER, a mammalian expression vector that directs the synthesis of siRNAs (Brummelkamp et al., 2002) was digested with both *Xho*l I and *Hin*dIII. Annealing shRNA was cloned into *Xho*l I and *Hin*dIII-digested pSUPER (pSUP): ${\text{pSUPER - siI}}_{2}^{\text{PP2A}}\;\left({\text{pSUP - siI}}_{2}^{\text{PP2A}}\right)$ and pSUPER-siCon (pSUP-siC).

### Production of lentiviral vectors

Vector plasmids were constructed for the production of third-generation lentivirus-expressing siRNA for ${\text{I}}_{2}^{\text{PP2A}}$. Fortunately, the siRNA target for human ${\text{I}}_{2}^{\text{PP2A}}$ also paired with the sequence of mouse ${\text{I}}_{2}^{\text{PP2A}}$ and knockdown ${\text{I}}_{2}^{\text{PP2A}}$ level in N2a cells. All vectors contained the eGFP coding sequence located in the middle of the lentiviral vector. This sequence is driven by a cytomegalovirus (CMV) promoter and terminates using the polyadenylation signal in the 3′ long terminal repeat (LTR). Downstream of the eGFP is a woodchuck hepatitis virus regulatory element (WPRE) that enhances the expression of the transgene. Recombinant lentiviruses were produced by transient transfection in HEK293T cells using the calcium phosphate transfection method, as described previously (Naldini et al., 1996). The infectious lentiviruses were harvested at 48 and 72 h post-transfection and filtered through 0.22-μm-pore cellulose acetate filter. The infectious lentiviruses were concentrated by ultracentrifugation (2 h at 50,000 × *g*) and subsequently purified by ultracentrifugation on a 20% sucrose gradient (2 h at 46,000 × *g*) as described (Naldini et al., 1996). Vector concentrations were analyzed using an immunocapture p24-gag ELISA (Alliance; DuPont-NEN; Naldini et al., 1996) and by flow cytometry quantification of eGFP-positive transduced cells, as described previously (Marr et al., 2003).

### Brain injection of ${\text{Lenti - siI}}_{2}^{\text{PP2A}}$

The human tau transgenic mice (htau, ~11-month-old) [STOCK *Mapt^tm1(EGFP)Klt^* Tg(MAPT)8cPdav/J, Jackson Lab], which express six isoforms of tau and show an age-dependent development tau pathology and impairments of cognitive and synaptic functions (Polydoro et al., 2009) were used for the study. For brain injections, the mice were positioned in a stereotaxic instrument and 2 μl ${\text{Lenti - siI}}_{2}^{\text{PP2A}}$ or ${\text{Lenti - ssiI}}_{2}^{\text{PP2A}}$ were injected into the hippocampus (AP – 2.0, ML – 1.5, DV – 2.0; Kaspar et al., 2002) at a rate of 0.50 μl/min. The syringe was left in the place for ~3 min before being slowly withdrawn from the brain. After 4 weeks, the mice were sacrificed, and the hippocampi were quickly removed out and homogenized on ice in lysis buffer [50 mM Tris–HCl (pH 7.5), 150 mM NaCl, 1% (v/v) Triton X-100, 1% (w/v) deoxycholate, 0.1% (w/v) SDS, 10 mM NaF, 1 mM Na_3_VO_4_, and 2 μg/ml each of aprotinin, leupeptin, and pepstatin A], then brain extracts stored in −80°C. All mice were kept at 24 ± 2°C on daily 12 h light–dark cycles with *ad libitum* access to food and water. The animal experiments were carried out according to the “Policies on the Use of Animals and Humans in Neuroscience Research” approved by the Society for Neuroscience in 1995, and also approved by Institutional Animal Care and Use Committee at Tongji Medical College, Huazhong University of Science and Technology.

### Step-down avoidance test

Four weeks after the brain infusion of the lentiviral vectors, the step-down avoidance test was performed by following a previous procedure (Zarrindast et al., 2006). Briefly, the apparatus consisted of an open field gray Plexiglas box (40 cm × 40 cm) with a steel rod floor. The Plexiglas platform (4 cm × 4 cm × 4 cm) was set in the center of the grid floor. Intermittent electric shocks (20 mA, 50 Hz) were delivered to the grid floor by an isolated stimulator. On the first day, each mouse was gently placed on the platform. When the mouse stepped down from the platform and placed all its paws on the grid floor, an intermittent electric shock was delivered for 3 s. Responsiveness to the punishment in the training test was assessed by the animal’s vocalization, only those mice that vocalized touching the grid with the four paws were used for the retention test in order to exclude the mice with a different pain threshold. Two hours [short-term memory (STM)] or 24 h [(long-memory (LTM)] after training, each mouse was placed on the platform again. The first time spent before stepping down onto the grid (latency period) and frequency (number of errors) stepping down the platform were measured, considering 300 s as the upper cut-off, during the training and retention tests.

### Cell culture and transient expression

The human embryonic kidney 293 cells or mouse N2a neuroblastoma cells stably expressing the longest human tau (tau441) cDNA (HEK293/tau or N2a/tau) were cultured in DMEM supplemented with 10% fetal bovine serum (FBS). The cells were maintained at 37°C in 5% CO_2_. The cells were plated onto six-well plates overnight and pSUP, pSUP-siC, or ${\text{pSUP - siI}}_{2}^{\text{PP2A}}$ plasmid was transfected the next day using Lipofectamine 2000 according to the manufacturer’s instruction.

### Activity assay of protein kinases PP2A

The cells were transfected with pSUP, pSUP-siC, or ${\text{pSUP - siI}}_{2}^{\text{PP2A}}$ plasmids. After 24 h, the cell lysate was prepared by adding lysis buffer [20 mM MOPS, 50 mM β-glycerophosphate, 50 mM sodium fluoride, 1 mM sodium vanadate, 5 mM EGTA, 2 mM EDTA, 1% NP40, 1 mM dithiothreitol (DTT), 1 mM benzamidine, 1 mM phenylmethanesulfonyl fluoride (PMSF), and 10 μg/ml leupeptin and aprotinin, pH 7.2]. The activity of PKA in the extract was assayed using a PKA kinase activity assay kit (Assay Designs, Inc.,) according to the manufacturer’s protocol.

The activity of PP2A was assayed using a serine/threonine phosphatase assay kit (Promega, MA, USA). The assay was based on determining the amount of free phosphate generated in the reaction by measuring the absorbance of a molybdate malachite green–phosphate complex. Cell extracts were prepared as follows: cells were rinsed twice with ice-cold phosphate-buffered saline and then scraped into 1 ml of ice-cold phosphatase buffer [50 mM Tris, pH 7.0, 0.1 mM ethylenediaminetetraacetic acid/ethylene glycol-*bis*(β-aminoethyl ether)-*N*,*N*,*N*′,*N*′-tetraacetic acid, 1 mM DTT, 0.1% (v/v) Triton X-100, benzamide, leupeptin, 4-(2-aminoethyl) benzene-sulfonyl fluoride⋅HCl (AEBSF), and pepstatin A]. The resulting cell suspension was lysed by brief sonication and cell debris were pelleted at 15,000 × *g* for 30 min. Free intracellular phosphate and ATP were removed from this resulting supernatant in a spin column containing Sephadex G-25 according to the supplier’s instructions. The sample (10 μg) was incubated on a 96-well plate together with a peptide substrate RRA(pT)VA and PP2A-specific reaction buffer (50 mM imidazole, pH 7.2, 0.2 mM EGTA, 0.02% β-mercaptoethanol, 0.1 mg/ml BSA) for 30 min at 30°C. After incubation, the molybdate complex dye was added and incubated for an additional 30 min at room temperature for color development. The level of molybdate malachite green–phosphate complex formed was monitored at 630 nm.

### Golgi staining

Golgi staining was performed according to methods as followed (Woolley and McEwen, 1992). The mice (*n* = 3 per group) were killed by overdose of chloral hydrate, and perfused through the aorta with 200 ml 0.9% NaCl containing 0.5% sodium nitrite followed by 500 ml 0.9% NaCl containing 5% formaldehyde. Then, the brain was fixed *in situ* by perfusion of Golgi fixative (0.9% NaCl, 5% formaldehyde, 5% potassium dichromate, 5% chloral hydrate) in the dark. The brain was removed and processed for rapid Golgi staining in the dark. Briefly, the brain was post-fixed for 3 days in the same Golgi fixative, and impregnated with 1.0% aqueous silver nitrate solution for 3 days. Coronal brain sections of hippocampal tissue were cut at 35 μm using a vibratome (VT1000S, Leica, Germany). The images were observed by using a microscope (Olympus BX60, Tokyo, Japan). Neurons in the CA3 region which fulfill the following criteria were selected for the analysis; (i) the cell type must be identifiable, (ii) image resolution should be sufficient to visually distinguish dendritic spine formation from variably contrasting background, and (iii) completeness of Golgi impregnation of all dendrites. Subjective bias in spine counting was eliminated by prior coding of slides.

To analyze the dendritic morphology, *Z*-stacks (step size 1 μm) from five to seven cells were generated using a confocal microscope (LSM510, Zeiss) in bright-field mode (20× objective) and reconstructed in ImagePro in combination with the NeuroDraw toolbox for each animal. Total dendritic length and number of branch points were analyzed using NeuroExplorer software (MBF Bioscience, Williston, VT, USA).

To acquire images for spine analysis, the dendritic segments were imaged under bright-field illumination on a Zeiss Axio imager microscope with a 63× oil immersion objective, and spine morphology was analyzed according to a previously reported method (Magarinos et al., 2011), which does not assess spine density in a three dimensional manner but focuses on spines paralleled to the plane of section. Although the method may underestimate the total number of spines, it facilitates a direct comparison of treatment groups when they are analyzed in an identical manner. ImageJ software was used to calculate linear spine density (Spires-Jones et al., 2011), which was presented as the number of spines per 10 mm of dendrite length. The spine density was determined in two segments of dendrites at a distance of 90–110 μm (proximal) and 190–210 μm (distal) from the soma. From each animal, four neurons were selected from one slide, accounting for 36 neurons/per animal.

### Real-time PCR

Total RNA was isolated by using Trizol™(Invitrogen Life Technologies, Carlsbad, CA, USA) according to manufacturer’s instruction. Then total RNA (3 μg in 25 μl) was reversely transcribed and the produced cDNA (1 μl) was used to detect the transcripts. Real-time polymerase chain reaction (PCR) to determine gene copy number was performed using the Rotor-Gene 3000 Real-Time PCR Detection System (Corbett Research, Sydney, NSW, Australia) with the SYBR^®^ Premix Ex Taq™[Takara Biotechnology (Dalian) Co., Ltd., Dalian, China]. The expression level of GADPH housekeeping gene was used for normalization of GSK-3β mRNA expression level. Forward primer 5′-ACGCTCCCTGTGATTTATG-3′ and reverse primer 5′-CAAGAGGTTCTGCGGTTTA-3′ for GSK-3β; forward primer 5′-CTTCAACTCTGGTCAAATAATGCA-3′ and reverse primer 5′-GAACAAAAATATAACAAACTCCGC-3′ for ${\text{I}}_{2}^{\text{PP2A}}$; forward primer 5′-GAAGGTGAAGGTCGGAGTC-3′ and reverse primer 5′-GAAGATGGTGATGGGATTTC-3′ for GADPH.

### Western blotting

Western blotting was performed according to methods established in our laboratory. Briefly, the cell homogenates or brain extracts were mixed with sample buffer containing 50 mM Tris–HCl (pH 7.6), 2% SDS, 10% glycerol, 10 mM DTT, and 0.2% bromophenol blue and boiled for 5 min. The proteins were separated by 10% SDS/PAGE and transferred to PVDF membrane. Immunostaining was visualized with a chemiluminescent substrate kit and CL-XPosure Film and quantitatively analyzed by digital science 1D software (Eastman Kodak, Rochester, NY, USA). Band intensity was measured as the sum optical density and expressed as a level relative to each control. The phosphorylated levels of tau were normalized relative to the total tau.

### Immunofluorescence staining

The transgenic mice were sacrificed by overdose chloral hydrate (1 g/kg) after injection of lentiviral vectors for about 1 month, and perfused through aorta with 100 ml 0.9% NaCl followed by 400 ml phosphate buffer containing 4% paraformaldehyde. Brains were removed and post-fixed in perfusate overnight and then cut into sections (20 μm) with a vibratome (Leica, Nussloch, Germany; S100, TPI). Sections were incubated at 4°C overnight with primary antibodies (see Table S1 in Supplementary Material and Figure legends). The images were observed using a laser scanning confocal microscope (Olympus FV500, Tokyo, Japan).

For cell studies, cells were cultured on coverslips and fixed with 4% paraformaldehyde for 1.5 h at 4°C and then incubated for 12–36 h at 4°C with primary antibodies overnight as indicated in each figure, and the immunoreactivity was probed with rhodamine red X- or Oregon green 488-conjugated secondary antibodies (see Table S1 in Supplementary Material).

### Statistical analysis

The data were expressed as mean ± SD and analyzed by the one-way analysis of variance procedure followed by least significant difference *post hoc* tests or Student’s *t*-tests for three groups, and Student’s *t*-test for two groups using SPSS 12.0 statistical software (SPSS Inc., Chicago, IL, USA). A *p* value of <0.05 was considered as statistically significant in all experiments.

## Results

### Silencing ${\text{I}}_{2}^{\text{PP2A}}$ attenuates tau hyperphosphorylation with improvement of memory deficits in htau transgenic mice

**Figure 1:**
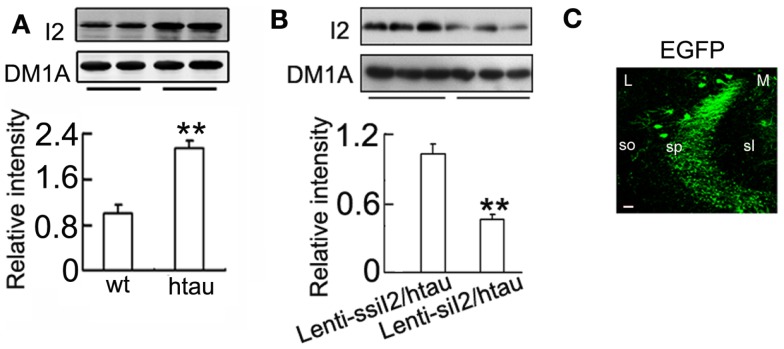
**Expression of ${\text{Lenti - siI}}_{2}^{\text{PP2A}}$ decreases ${\text{I}}_{2}^{\text{PP2A}}$ level in htau mice**. **(A)** ${\text{I}}_{2}^{\text{PP2A}}$ (I_2_) protein in the hippocampus of the ~11-month-old htau mice or the age-control wild-type (wt) mice was detected by Western blotting. **(B)** Approximately 11-month-old htau mice received brain infusion of the ${\text{Lenti - siI}}_{2}^{\text{PP2A}}$ (Lenti-siI_2_) or the scrambled ${\text{Lenti - ssiI}}_{2}^{\text{PP2A}}$ (Lenti-ssiI_2_; 2 μl each, 2 × 10^9^ TU/ml) into the hippocampal CA3 region under a stereotaxic instrument as described in the Section “Materials and Methods.” Four weeks later, the expression levels of ${\text{I}}_{2}^{\text{PP2A}}$ were detected by Western blotting and quantitative analysis. **(C)** The expression of the lentivirus in CA3 region was presented. The eGFP was stained in the cell body and dendrites of neurons. L, lateral; M, medial; sl, stratum lucidum; so, stratum oriens; sp, stratum pyramidale. The data were presented as mean ± SD of three independent experiments. **p < 0.01 vs. wt mice or Lenti-ssiI_2_/htau2 (htau mice infused with Lenti-ssiI_2_). Scale bar: 20 μm.

**Figure 2:**
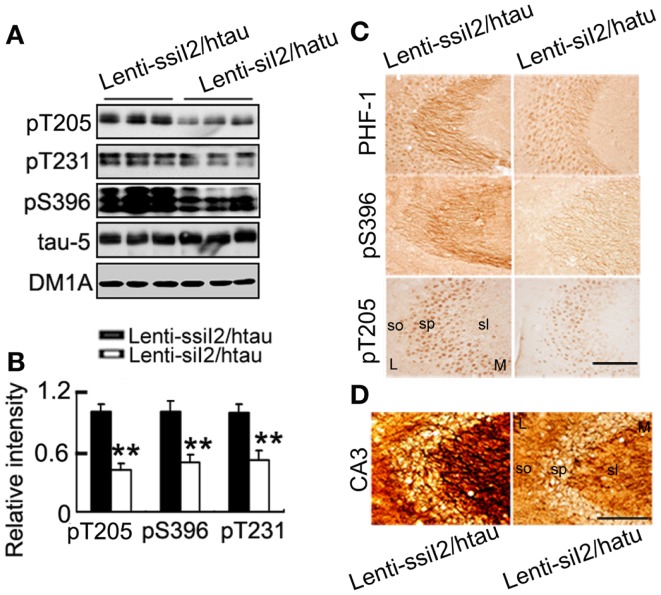
**Expression of ${\text{Lenti - siI}}_{2}^{\text{PP2A}}$ arrests tau pathology in htau mice**. Approximately 11-month-old htau mice received brain infusion of the ${\text{Lenti - siI}}_{2}^{\text{PP2A}}$ (Lenti-siI_2_) or the scrambled ${\text{Lenti - ssiI}}_{2}^{\text{PP2A}}$ (Lenti-ssiI_2_; 2 μl each, 2 × 10^9^ TU/ml) into the hippocampal CA3 region under a stereotaxic instrument as described in the Section “Materials and Methods.” Four weeks later, the expression level of tau phosphorylation at several AD-related sites was detected by Western blotting **(A,B)** and immunohistochemistry **(C)**. The silver staining images were represented. **(D)** L, lateral; M, medial; sl, stratum lucidum; so, stratum oriens; sp, stratum pyramidale. The data were presented as mean ± SD of three independent experiments. *p < 0.05; **p < 0.01 vs. Lenti-ssiI_2_/htau2 (htau mice infused with Lenti-ssiI_2_). Scale bars: 100 μm.

The transcription and expression of ${\text{I}}_{2}^{\text{PP2A}}$ is significantly increased in the AD brains (Tanimukai et al., 2005), and increasing ${\text{I}}_{2}^{\text{PP2A}}$ by AAV transfection in rat brain induced AD-like pathology and cognitive impairment (Wang et al., 2010). We found that ${\text{I}}_{2}^{\text{PP2A}}$ protein level was significantly increased in htau transgenic mice compared with the wild-type mice (Figure 1A), while intracranial injection of ${\text{Lenti - siI}}_{2}^{\text{PP2A}}$ into the hippocampus of htau transgenic mice, a recognized AD-like animal model for tau pathology (Polydoro et al., 2009), reduced the ${\text{I}}_{2}^{\text{PP2A}}$ level to ~45% of the control level at 4 weeks after the injection (Figure 1B). Simultaneously, the phosphorylation level of tau at Thr-205 (pT205), Thr-231 (pT231), Ser-396 (pS396), and Ser-396/404 (PHF-1) epitopes was significantly reduced compared with the htau mice injected with the scrambled ${\text{Lenti - siI}}_{2}^{\text{PP2A}}$ controls (Figures 2A–C). By silver staining, we observed that the accumulation of argyrophilic substances was also significantly decreased by expression of ${\text{Lenti - siI}}_{2}^{\text{PP2A}}$ (Figure 2D).

**Figure 3:**
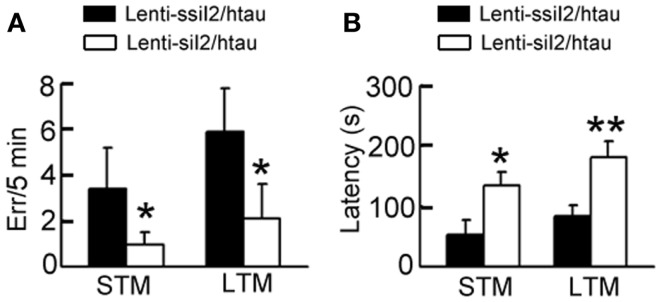
**Expression of ${\text{Lenti - siI}}_{2}^{\text{PP2A}}$ improves cognitive functions in htau mice**. Approximately 11-month-old htau mice received brain infusion of the ${\text{Lenti - siI}}_{2}^{\text{PP2A}}$ (Lenti-siI_2_) or the scrambled ${\text{Lenti - ssiI}}_{2}^{\text{PP2A}}$ (Lenti-ssiI_2_; 2 μl each, 2 × 10^9^ TU/ml) into the hippocampal CA3 region under a stereotaxic instrument. Four weeks later, the associative short-term memory (STM) **(A)** and long-term memory (LTM) **(B)** were detected by step-down avoidance test (*n* = 8–10 for each group). The data were expressed as mean ± SD. *p < 0.05; **p < 0.01 vs. Lenti-ssiI_2_/htau (htau mice infused with Lenti-ssiI_2_).

A previous study has demonstrated that the htau transgenic mice show learning and memory deficits at 12 months (Polydoro et al., 2009). By step-down avoidance test, we observed that silencing ${\text{I}}_{2}^{\text{PP2A}}$ improved both the STM and the LTM (Figure 3). These data demonstrate that silencing ${\text{I}}_{2}^{\text{PP2A}}$ by ${\text{Lenti - siI}}_{2}^{\text{PP2A}}$ could antagonize tau pathology and memory deficits in htau transgenic mice.

### Silencing ${\text{I}}_{2}^{\text{PP2A}}$ ameliorates dendrite complexity and spine density in htau mice

**Figure 4:**
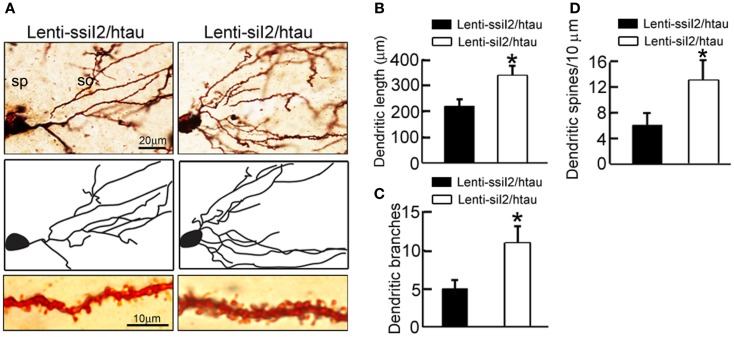
**Expression of ${\text{Lenti - siI}}_{2}^{\text{PP2A}}$ remodels the dendrite complexity in htau mice**. Approximately 11-month-old htau mice received brain infusion of the ${\text{Lenti - siI}}_{2}^{\text{PP2A}}$ (Lenti-siI_2_) or the scrambled ${\text{Lenti - ssiI}}_{2}^{\text{PP2A}}$ (Lenti-ssiI_2_; 2 μl each, 2 × 10^9^ TU/ml) into the hippocampal CA3 region under a stereotaxic instrument. Four weeks later **(A–D)**, representative Golgi stained hippocampal CA3 neurons with its corresponding morphological drawings **(A)** and quantitative analysis of the total dendrite length **(B)**, the number of dendritic branch **(C)**, and the density of dendritic spines **(D)**. So, stratum oriens; sp, stratum pyramidale. The data were expressed as mean ± SD. *p < 0.05; **p < 0.01 vs. Lenti-ssiI_2_/htau (htau mice infused with Lenti-ssiI_2_).

To explore the molecular bases underlying the improved memory by silencing ${\text{I}}_{2}^{\text{PP2A}}$, we analyzed the dendritic morphology and spine density of the neurons in hippocampal CA3 region of the htau transgenic mice using Golgi stain. The dendritic length and the number of branches were assessed as a measure of dendritic complexity. We found that silencing ${\text{I}}_{2}^{\text{PP2A}}$ increased the dendritic length, the number of branches, and the density of the dendritic spines (Figure 4), suggesting that silencing ${\text{I}}_{2}^{\text{PP2A}}$ promotes dendritogenesis.

### Silencing ${\text{I}}_{2}^{\text{PP2A}}$ inhibits GSK-3β with activation of PKA and Akt in htau transgenic mice

**Figure 5:**
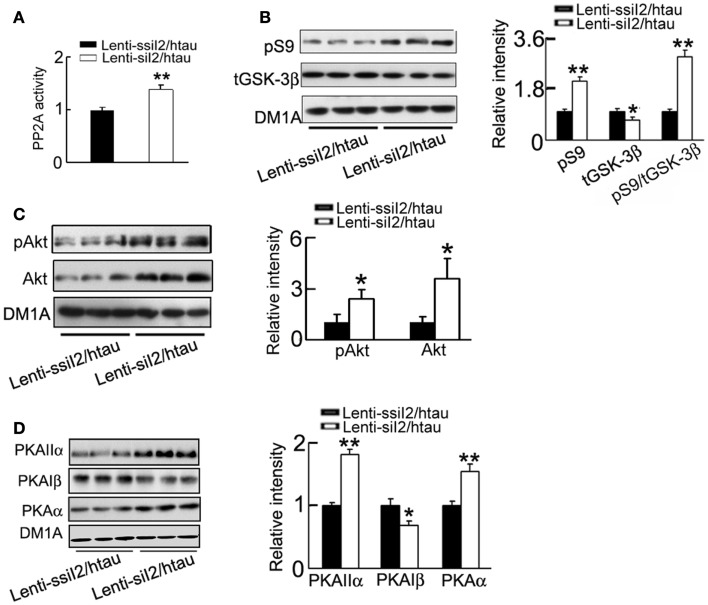
**Expression of ${\text{Lenti - siI}}_{2}^{\text{PP2A}}$ restores PP2A activity with inhibition of GSK-3 in htau mice**. Approximately 11-month-old htau mice were treated as Figure 1. **(A)** The activity of PP2A was measured by a PP2A activity assay kit as described in the Section “Materials and Methods.” **(B–D)** The protein levels of the Ser9-phosphorylated GSK-3β (pS9) and total GSK-3β (tGSK-3β) **(B)**, the levels of phosphorylated Akt at Ser473 (pS473-Akt) and total Akt **(C)**, and the levels of PKAα, PKAIβ, and PKAIIα **(D)** were measured by Western blotting and quantitative analysis. The data were presented as mean ± SD of three independent experiments. *p < 0.05; **p < 0.01 vs. Lenti-ssiI_2_/htau2 (htau mice infused with Lenti-ssiI_2_).

Inhibitor-2 of protein phosphatase-2A was originally identified to regulate PP2A (Li et al., 1995, 1996), therefore we first measured PP2A activity after silencing ${\text{I}}_{2}^{\text{PP2A}}$. As expected, silencing ${\text{I}}_{2}^{\text{PP2A}}$ significantly increased PP2A activity (Figure 5A). Our recent data show that knockdown of ${\text{I}}_{2}^{\text{PP2A}}$ decreases GSK-3β protein level in HEK293 cell lines (Liu et al., 2012), the most implicated tau kinase (Ishiquro et al., 1993; Takashima et al., 1996; Avila and Díıaz-Nido, 2004; Takashima, 2006), and thus we also measured the alteration of GSK-3β in hippocampus of the mice after silencing ${\text{I}}_{2}^{\text{PP2A}}$. We observed that silencing ${\text{I}}_{2}^{\text{PP2A}}$ could inhibit GSK-3β, demonstrated by the reduced total level of GSK-3β (tGSK-3β) and elevation of Ser9-phosphorylated GSK-3β (pS9-GSK-3β; Figure 5B). To further explore how GSK-3β activity is regulated, we measured the alteration of Akt and PKA, the known kinases regulating Ser9 phosphorylation of GSK-3β (Cross et al., 1995; Shaw et al., 1997; Fang et al., 2000). We found that silencing ${\text{I}}_{2}^{\text{PP2A}}$ activated Akt with increased levels of phosphorylated Akt at Ser473 epitope and total Akt, and PKA with increased levels of PKAα (catalytic subunit) and PKAIIα (regulatory subunit) and decreased PKAIβ (regulatory subunit) level in the hippocampus (Figures 5C,D).

### Silencing ${\text{I}}_{2}^{\text{PP2A}}$ inhibits GSK-3β through activation of PKA but not Akt demonstrated in HEK293/tau or N2a/tau cells

**Figure 6:**
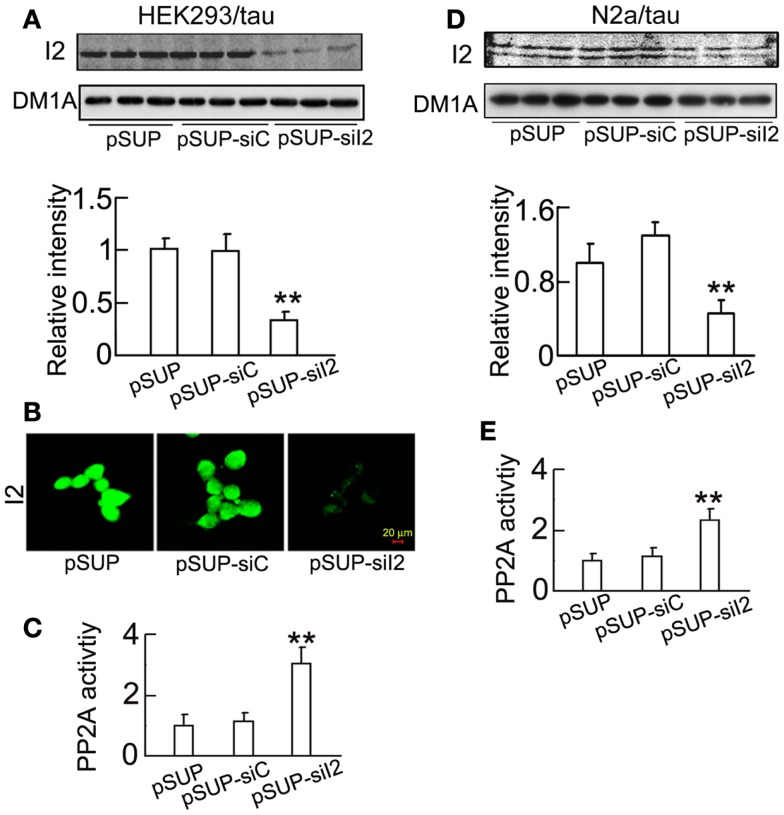
**${\text{SiI}}_{2}^{\text{PP2A}}$ decreases the expression of ${\text{I}}_{2}^{\text{PP2A}}$ and increases PP2A activity in cell lines**. ${\text{pSUP - siI}}_{2}^{\text{PP2A}}$ (pSUP-siI_2_) was transfected into HEK293/tau or N2a/tau cells to knockdown the expression of ${\text{I}}_{2}^{\text{PP2A}}$, pSUP, and pSUP-siC were transfected as the controls. After 24 h, the level of ${\text{I}}_{2}^{\text{PP2A}}$ in the cell extracts was estimated by Western blotting **(A,D)** and immunostaining **(B)**. The activity of PP2A was measured by a PP2A activity assay kit as described in the Section “Materials and Methods” **(C,E)**. The relative intensity was normalized against DM1A (to α-tubulin) and expressed by setting pSUP as 1. The data were presented as mean ± SD of at least three independent experiments. **p < 0.01 vs. pSUP.

**Figure 7:**
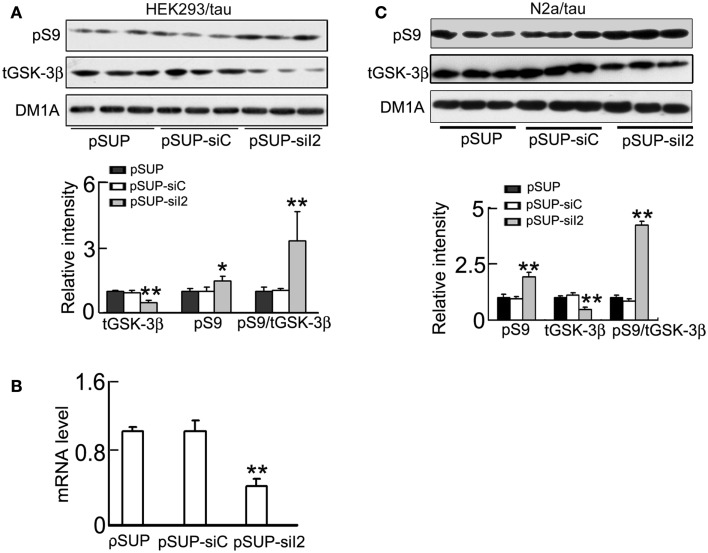
**Knockdown ${\text{I}}_{2}^{\text{PP2A}}$ phosphorylates GSK-3β at Ser9 and inhibits GSK-3β activity**. HEK293/tau or N2a/tau cells were transfected with ${\text{pSUP - siI}}_{2}^{\text{PP2A}}$ (pSUP-siI_2_) for 24 h, and pSUP and pSUP-siC were transfected as the controls. **(A,C)** The protein levels of total (tGSK-3β) and the Ser9-phosphorylated GSK-3β (pS9, the inactive form) were estimated by Western blotting normalized against DM1A. **(B)** The mRNA level of GSK-3β was analyzed by real-time PCR, normalized against GADPH and expressed by setting pSUP as 1. The data were presented as mean ± SD of three independent experiments. *p < 0.05; **p < 0.01 vs. pSUP.

To further verify the role of ${\text{I}}_{2}^{\text{PP2A}}$ silencing in the regulation of GSK-3β, we constructed ${\text{pSUP - siI}}_{2}^{\text{PP2A}}$ and transfected the plasmid into HEK293/tau or N2a/tau cells with pSUP (empty vector) and pSUP-siC (control vector of siRNA) as controls. As observed in htau transgenic mice, silencing ${\text{I}}_{2}^{\text{PP2A}}$ significantly decreased the ${\text{I}}_{2}^{\text{PP2A}}$ level in HEK293/tau cells (Figures 6A,B) and N2a/tau cells (Figure 6D). Simultaneously, the activity of PP2A was restored after ${\text{I}}_{2}^{\text{PP2A}}$ knockdown in HEK293/tau and N2a/tau cells (Figures 6C,E). ${\text{I}}_{2}^{\text{PP2A}}$ knockdown also down-regulated GSK-3β, demonstrated by the reduction of total GSK-3β (tGSK-3β) protein and mRNA levels, and elevation of the pS9-GSK-3β (the inactive form) in HEK293/tau cells (Figures 7A,B) and N2a/tau cells (Figure 7C). These *in vitro* data further confirm that silencing ${\text{I}}_{2}^{\text{PP2A}}$ not only activates PP2A but also inhibits GSK-3β.

**Figure 8:**
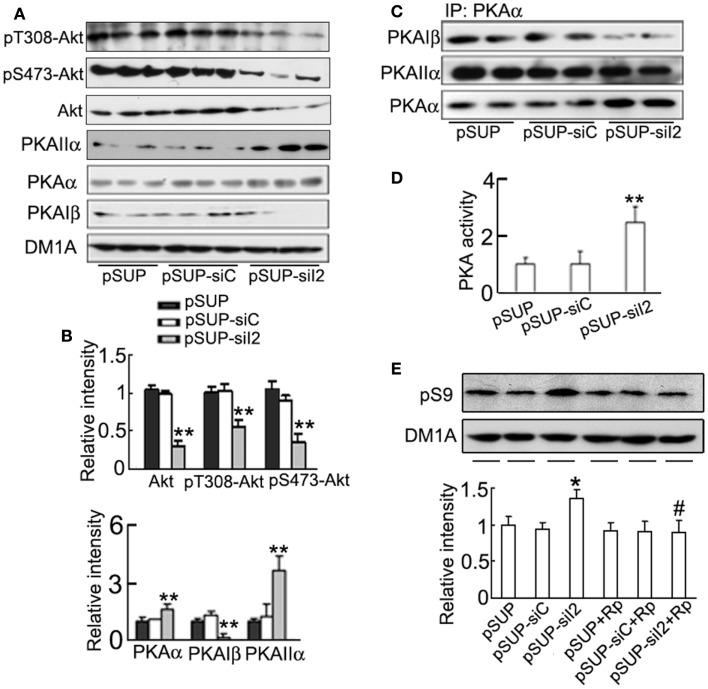
**Activation of PKA is responsible for the phosphorylation of GSK-3β at Ser9 induced by ${\text{I}}_{2}^{\text{PP2A}}$ knockdown**. HEK293/tau cells were transfected with ${\text{pSUP - siI}}_{2}^{\text{PP2A}}$ (pSUP-siI_2_) for 24 h, and pSUP and pSUP-siC were expressed as the controls. **(A,B)** The levels of phosphorylated Akt at Thr308, Ser 473, total Akt, PKAα, PKAIIα, and PKAIβ were estimated by Western blotting **(A)** and quantitative analysis **(B)**. **(C)** The cell lysates were subjected to immunoprecipitation (IP) with anti-PKAα antibody, and the precipitates were probed by anti-PKAα, anti-PKAIβ, or anti-PKAIIα. **(D)** The activity of PKA was also measured by a PKA assay kit as described in the Section “Materials and Methods.” **(E)** The cells with overexpression of pSUP-siI_2_, pSUP-siC, or the vector were treated with *R*_p_-cAMPS (*R*_p_, 10 μM) for 30 min, and then pS9-GSK-3β was detected by Western blotting and quantitative analysis. The relative intensity was normalized against DM1A and expressed by setting pSUP as 1. The data were presented as mean ± SD of three independent experiments. *p < 0.05;**p < 0.01 vs. pSUP; ^#^*p* < 0.05 vs. pSUP-siI_2_.

Akt and PKA are known kinases to regulate Ser9 phosphorylation of GSK-3β (Cross et al., 1995; Shaw et al., 1997; Fang et al., 2000). Therefore, we studied the role of Akt and PKA in phosphorylating (inhibiting) pS9-GSK-3β. We found unexpectedly that the total Akt level and the phosphorylated Akt at Thr308 and Ser473 (active form) decreased by silencing ${\text{I}}_{2}^{\text{PP2A}}$ (Figures 8A,B), suggesting that Akt activity was decreased by ${\text{I}}_{2}^{\text{PP2A}}$ knockdown. These data ruled out the role of Akt in phosphorylating GSK-3β during ${\text{I}}_{2}^{\text{PP2A}}$ knockdown. However, the catalytic subunit α (PKAα) and the regulatory subunit IIα (PKAIIα) of PKA increased, whereas the regulatory subunit Iβ (PKAIβ) of PKA decreased after silencing ${\text{I}}_{2}^{\text{PP2A}}$ in HEK293/tau cells (Figures 8A,B). It is known that PKA is activated when the catalytic subunit is released from the tetrameric holoenzyme, which is modulated by the binding capacity of catalytic subunit to the regulatory subunits. Therefore, we measured the interactions of PKAα with its regulatory subunits by co-immunoprecipitation assay. The results showed that the association level of PKAα with PKAIβ decreased, whereas the binding of PKAα with PKAIIα was not altered after ${\text{I}}_{2}^{\text{PP2A}}$ knockdown (Figure 8C). To confirm the alteration of PKA activity, we used ELISA assay, and a significantly increased PKA activity was detected by ${\text{I}}_{2}^{\text{PP2A}}$ knockdown (Figure 8D). These data indicate that silencing ${\text{I}}_{2}^{\text{PP2A}}$ can also activate PKA.

To further verify the role of PKA in GSK-3β inhibition induced by silencing ${\text{I}}_{2}^{\text{PP2A}}$, we used *R*_p_-cAMPS, a specific inhibitor of PKA, after transfection of ${\text{siI}}_{2}^{\text{PP2A}}$. We found that simultaneous application of *R*_p_-cAMPS abolished the ${\text{siI}}_{2}^{\text{PP2A}}$-induced inhibitory phosphorylation of GSK-3β at Ser9 (Figure 8E).

**Figure 9:**
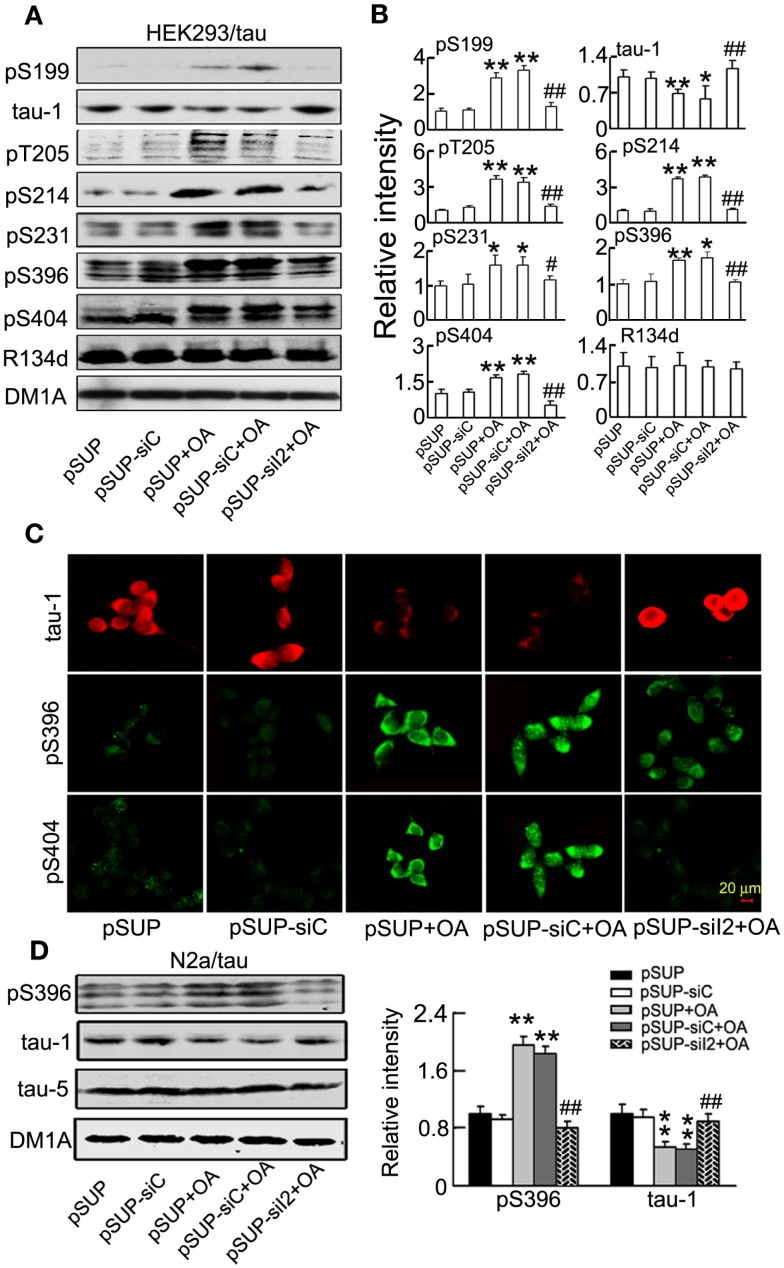
**${\text{siI}}_{2}^{\text{PP2A}}$ attenuates tau hyperphosphorylation induced by okadaic acid in HEK293/tau or N2a/tau cells**. ${\text{pSUP - siI}}_{2}^{\text{PP2A}}$ (pSUP-siI_2_) was transfected into HEK293/tau **(A–C)** or N2a/tau cells **(D)** to knockdown the expression of ${\text{I}}_{2}^{\text{PP2A}}$, and pSUP and pSUP-siC were expressed as the controls. After 24 h, OA (okadaic acid, 25 nM) was administered for 24 h. **(A,B)** The relative levels of hyperphosphorylated tau at Ser-199 (pS199), Ser-198/202 (tau-1), Thr-205 (pT205), Ser-214 (pS214), Thr-231 (pT231), Ser-396 (pS396), and Ser-404 (pS404) epitopes were detected by Western blotting **(A)** and quantitative analysis **(B)**. **(C)** The phosphorylated levels of tau at Ser-198/202 (tau-1), Ser-396, and Ser-404 epitopes were detected by immunofluorescence staining. **(D)** The relative levels of hyperphosphorylation tau at Ser-198/202 (tau-1) and Ser-396 (pS396) epitopes were detected by Western blotting and quantitative analysis. The relative intensity was normalized against total tau probed by R134d or tau-5 and expressed by setting pSUP as 1. DM1A serves as a loading control. The data were presented as mean ± SD of at least three independent experiments. *p < 0.05; **p < 0.01 vs. pSUP; ^#^*p* < 0.05; ^##^*p* < 0.01 vs. pSUP + OA.

We also studied whether silencing ${\text{siI}}_{2}^{\text{PP2A}}$ could attenuate tau hyperphosphorylation induced by OA (PP2A inhibitor) *in vitro*. We transfected ${\text{pSUP - siI}}_{2}^{\text{PP2A}}$ into HEK293/tau or N2a/tau cells, and treated the cells with OA (25 nM) for 24 h. Then, we detected tau phosphorylation by Western blotting. We found that silencing ${\text{I}}_{2}^{\text{PP2A}}$ significantly reduced tau phosphorylation at Ser-199 (pS199), Thr-205 (pT205), Ser-214 (pS214), Ser231 (pS231), Ser-396 (pS396), and Ser-404 (pS404), and increased the level of the unphosphorylated tau at Ser-198/202 (tau-1) induced by OA in HEK293/tau (Figures 9A,B). The immunofluorescence data confirmed the same results (Figure 9C). We also found that knockdown ${\text{I}}_{2}^{\text{PP2A}}$ significantly decreased tau phosphorylation induced by OA in N2a/tau cells (Figure 9D).

## Discussion

As the name designated, ${\text{I}}_{2}^{\text{PP2A}}$ was originally identified as endogenous protein inhibitor of PP2A. We noticed in a recent study that ${\text{I}}_{2}^{\text{PP2A}}$ may also regulate GSK-3 (Liu et al., 2012). In the present study, we demonstrated that knockdown ${\text{I}}_{2}^{\text{PP2A}}$ could inhibit GSK-3 activity by decreasing the levels of GSK-3β mRNA and protein with an increased inhibitory phosphorylation of GSK-3β at Ser9 in HEK293/tau and N2a/tau cells, and htau transgenic mice. Our data provide the first evidence that knockdown ${\text{I}}_{2}^{\text{PP2A}}$ not only restitutes PP2A, but also inhibits GSK-3β.

Glycogen synthase kinase-3β is the downstream of Akt signaling pathway and its N-terminal serine-9 residue can be phosphorylated by Akt (Cross et al., 1995; Shaw et al., 1997; Zhou et al., 2013). To explore the kinase(s) that may be responsible for the increased phosphorylation of GSK-3 at Ser9 by ${\text{I}}_{2}^{\text{PP2A}}$ knockdown, we firstly detected the activity of Akt. We observed that the Akt activity was decreased in HEK293/tau cell lines; however Akt activity increased in htau transgenic mice after ${\text{I}}_{2}^{\text{PP2A}}$ knockdown. The mechanism underlying this discrepancy is currently not understood. It is possible that in native neurons more signal transduction pathways are active compared to cell lines. Recent studies suggest that PKA can also phosphorylate and inactivate GSK-3β (Fang et al., 2000; Zhou et al., 2013), therefore, we detected the activity of PKA. We found that PKA was activated by ${\text{I}}_{2}^{\text{PP2A}}$ knockdown, whereas simultaneous inhibition of PKA by *R*_p_-cAMPs abolished the ${\text{I}}_{2}^{\text{PP2A}}$ knockdown-induced GSK-3β phosphorylation, suggesting that ${\text{I}}_{2}^{\text{PP2A}}$ knockdown may inhibit GSK-3β *via* activating PKA. The holoenzyme of PKA is composed of catalytic subunit of PKAα, and regulatory subunits PKAIIα and PKAIβ, and association of PKAα with PKAIIα and PKAIβ inhibits the activity of kinase. Therefore, we detected the expression levels of PKAα, PKAIIα, and PKAIβ and their interactions. Although ${\text{I}}_{2}^{\text{PP2A}}$ knockdown increased PKAIIα level that should be inhibitory, the association of PKAα with PKAIIα or PKAIβ was decreased, which supports the activation of PKA activity by ${\text{I}}_{2}^{\text{PP2A}}$ knockdown. Based on our findings, Akt seems not responsible for the *in vitro* GSK-3β inhibition in the cell lines, however, the *in vivo* role of Akt in GSK-3β inhibition may be relatively more important than PKA.

Several groups have reported that GSK-3β seems essential for cognitive function using genetically engineered mouse models. For instance, long-term potentiation inhibits the induction of long-term depression via activation of the PI3K–Akt–GSK-3β pathway (Peineau et al., 2007; Bradley et al., 2012), and heterozygote GSK-3β knockout mice show an impaired long-term memory formation and reconsolidation (Kimura et al., 2008). GSK-3β activities were far lower than normal levels in these studies. Increased GSK-3 activity is believed to play a key role in the pathogenesis of CNS chronic disorders such as AD and schizophrenia (Emamian et al., 2004; Engel et al., 2006). In AD, there are studies supporting GSK-3 activity increased (Hye et al., 2005; Leroy et al., 2007), and the activated GSK-3β is accumulated in a subpopulation of neurons with NFTs in the AD brains (Pei et al., 1999). GSK-3β was the first identified tau kinase, which can phosphorylate tau at most of the hyperphosphorylated sites seen in the PHFs isolated from AD brains (Lovestone et al., 1994; Hong et al., 1997; Lucas et al., 2001; Hernandez et al., 2013). In addition to phosphorylate tau, GSK-3β has been linked to all of the primary abnormalities associated with AD. These include interactions between GSK-3β and components of the plaque-producing amyloid system (Takashima et al., 1993; Aplin et al., 1996), and interactions of GSK-3β with presenilin (Takashima et al., 1998; Gantier et al., 2000; Dolma et al., 2014) and other AD-associated proteins (Grimes and Jope, 2001; Hohman et al., 2014). Therefore, decreasing GSK-3β activity may be potential for AD therapy (Engel et al., 2006; Gómez-Sintes et al., 2011). Whether the inhibition of GSK-3β by ${\text{I}}_{2}^{\text{PP2A}}$ knockdown may improve the cognition of the mice deserves further investigations.

Microtubule associated protein tau is a major cytoskeletal protein that regulates the dynamic structure and function of the neurons. As a major protein component of the NFTs that is positively correlated with the dementia in AD patients (Avila et al., 2002), and hyperphosphorylation of tau is a recognized factor contributing to the memory deficits. The abnormally hyperphosphorylated tau impairs the axonal transport (Terwel et al., 2005; Bertrand et al., 2013). In the present study, we also found that knockdown ${\text{I}}_{2}^{\text{PP2A}}$ improved the dendrite complexity and spine density, which may at least contribute to improved cognition.

Taken together, we have found in the present study that downregulation of ${\text{I}}_{2}^{\text{PP2A}}$ not only restitutes PP2A activity but also inhibits GSK-3β, which makes ${\text{I}}_{2}^{\text{PP2A}}$ a promising target to arrest AD-like tau hyperphosphorylation, and restores dendrite complexity and ameliorates cognitive deficits.

## Conflict of Interest Statement

The authors declare that the research was conducted in the absence of any commercial or financial relationships that could be construed as a potential conflict of interest.

## Supplementary Material

The Supplementary Material for this article can be found online at http://www.frontiersin.org/Journal/10.3389/fnagi.2014.00123/abstract

## Abbreviations

AD, Alzheimer’s disease; GSK-3β, glycogen synthase kinase-3β; ${\text{I}}_{1}^{\text{PP2A}}$, PP2A inhibitor 1; ${\text{I}}_{2}^{\text{PP2A}}$, inhibitor-2 of protein phosphatase-2A; NFTs, neurofibrillary tangles; OA, okadaic acid; PP2A, protein phosphatase-2A; WT, wortmannin.
