# Supplementary material for: Silencing I2PP2A Rescues Tau Pathologies and Memory Deficits through Rescuing PP2A and Inhibiting GSK-3β Signaling in Human Tau Transgenic Mice
Source: Front Aging Neurosci. 2014 Jun 17;6:123. doi: 10.3389/fnagi.2014.00123 (PMC4060416; doi:10.3389/fnagi.2014.00123)
Supplement: Supplementary file 1 [file DataSheet_1.PDF]

Supplementary Table 1 Antibodies emploied in the study

Antibody	Specific	Type	WB	IH	Source
$I_2^{PP\text{-}2A}$	Total inhibitor-2 of PP-2A	pAb	1:1500	1:200	Santa Cruz
$PP\text{-}2A_C$	PP-2A catalytic subunit (both α and β isoforms)	pAb	1:1000		Cell Signaling
pS9-GSK-3β	Phospho-GSK-3β (Ser9)	pAb	1:1000		Cell Signaling
GSK-3β	Total GSK-3β	pAb	1:1000		Cell Signaling
Akt	Total Akt	pAb	1:1000		Santa Cruz
pT308-Akt	Phosphorylated Akt at Thr308	pAb	1:1000		Cell Signaling
pS473-Akt	Phosphorylated Akt at Ser473	pAb	1:1000		Cell Signaling
PKA α	PKAα catalytic subunit	pAb	1:1000		SAB
PKA Iβ	PKA type Iβ regulatory subunit	pAb	1:1000		Santa Cruz
PKA IIα	PKA IIα, and to a lesser extent PKA IIβ regulatory subunit	pAb	1:1000		Santa Cruz
pS199	Phosphorylated tau at Ser^{199}	pAb	1:1000		Iqbal
Tau-1	Nonphosphorylated tau at Ser^{198}–Ser^{202}	mAb	1:1000	1:200	Chemicon
pT205	Phosphorylated tau at Thr^{205}	pAb	1:1000	1:200	SAB
pS214	Phosphorylated tau at Ser^{214}	pAb	1:1000		SAB
pT231	Phosphorylated tau at Thr^{231}	pAb	1:1000		SAB
pS396	Phosphorylated tau at Ser^{396}	pAb	1:1000	1:200	Biosource
PHF-1	Phosphorylated tau at Ser^{396}- Thr^{404}	pAb		1:200	Biosource
pT404	Phosphorylated tau at Thr^{404}	pAb	1:1000	1:200	SAB
Tau-5	Total tau	mAb	1:1000		SAB
R134d	Total tau	pAb	1:1000		Iqbal
DM1A	α-tubulin	mAb	1:2000		Sigma
